# Nanoindentation Crack Suppression and Hardness Increase in SrTiO_3_ by Dislocation Engineering

**DOI:** 10.1007/s11837-025-07148-x

**Published:** 2025-01-27

**Authors:** Jiawen Zhang, Oliver Preuß, Xufei Fang, Wenjun Lu

**Affiliations:** 1 Shenzhen Key Laboratory of Intelligent Robotics and Flexible Manufacturing Systems, Department of Mechanical and Energy Engineering, Southern University of Science and Technology, Shenzhen, 518055 China; 2 Department of Materials and Earth Sciences, Technical University of Darmstadt, Peter-Grünberg-Str. 2, 64287 Darmstadt, Germany; 3 Institute for Applied Materials, Karlsruhe Institute of Technology, Kaiserstrasse 12, 76131 Karlsruhe, Germany

## Abstract

Dislocations in functional oxides have sparked interest in the potential they hold for harvesting both enhanced mechanical and functional properties for next-generation electronic devices. This has motivated the recent research endeavor to achieve tunable dislocation density and plastic zone size in functional oxides. However, the dislocation density-dependent micro-/nanomechanical properties in functional ceramics have yet to be assessed, which will be critical for the design of reliable electronic devices in the near future. In this work, we use a model material, single-crystal SrTiO_3_, as one of the most widely used substrates for oxide electronics, to assess the hardness and fracture behavior at micro-/nanoscale by pre-engineering the dislocation densities from ~ 10^10^ m^-2^ up to ~ 4.0 × 10^14^ m^-2^. We find crack suppression and enhanced hardness during nanoindentation in samples with pre-engineered dislocations. Post-indentation analysis using transmission electron microscopy revealed the critical role of pre-existing dislocations in regulating the crack suppression and increased hardness in SrTiO_3_. The results can help guide the design of mechanically reliable electronics via dislocation engineering.

## Introduction

Oxide ceramics are often used in electronic devices or as substrates for growing thin films. The operation reliability of such devices is closely related to their mechanical properties.^1^ However, the room-temperature brittleness of functional oxides tends to cause failure by catastrophic fracture, which is a common concern for most oxide electronics. Surprisingly, dislocations in functional oxides, which have emerged in recent years, have shown enhanced mechanical properties, including toughness and plasticity,^2,3^ as well as functionalities, including conductivity,^4^ ferroelectric properties,^5^ and photoelectric properties.^6^ This contrasts the conventional picture that dislocations are not abundant in oxide ceramics and brings a new perspective to the design of *ductile* and functional electronic devices. Nonetheless, the intrinsic brittleness of most oxide ceramics leads to low fracture toughness (~ 0.1 to ~ 10 MPa·m^0.5^) for easy crack propagation compared to metals.^7^ In oxide ceramics, the dislocation density in pristine or conventionally sintered materials is much lower (~ 10^9^ m^-2^ to ~ 10^10^ m^-2^^8^) than in metals (~ 10^13^ m^-2^ to ~ 10^14^ m^-2^^9^). Moreover, it is widely believed that the strong ionic/covalent bonding hinders dislocation mobility. Realizing dislocation-based toughening and crack suppression in functional oxide ceramics has therefore been a long-standing challenge.

To achieve dislocation-based toughening in ceramics, crack-tip plasticity, namely, crack-tip dislocation emission, blunting, and/or dislocation shielding is required.^10^ The earlier studies mainly focused on materials with a certain degree of room-temperature plasticity with rock salt structures. For instance, using *in situ* straining in a high-voltage transmission electron microscope, Appel et al. found that dislocation enrichment in front of the crack tip offers a plastic zone in MgO.^11^ In NaCl, it was reported that crack-tip shielding with dislocations can lead to a higher fracture toughness (~ 1.4 times increase).^12^ However, crack-tip dislocation emission in most ceramics (and other brittle materials) is not readily accessible at room temperature because of the extremely high energy barrier for dislocation nucleation. As an alternative, we propose to first pre-engineer high-density dislocations to circumvent the dislocation nucleation. As demonstrated most recently, it was found that with pre-engineered high-density dislocations (~ 4 × 10^15^ m^-2^) in rock-salt MgO ceramics, the damage tolerance and Vickers indentation fracture resistance were greatly improved, with even complete crack suppression by room-temperature Vickers indentation up to a load of 0.1 kg.^10^

Similarly, in perovskite oxides, the enrichment of pre-existing dislocations also leads to increased fracture resistance. Dislocation enrichment in (110) SrTiO_3_ yields an increased crack-tip toughness from 0.75 MPa·m^0.5^ to 1.60 MPa·m^0.5^.^2^ Using Brinell ball cyclic indentations, then combined with thermal treatment, Negm et al. reported both enhanced indentation fracture resistance and microhardness in (001) SrTiO_3_ single crystal owning to the increased dislocation density, where the indentation fracture resistance was improved ~ 20% as the dislocation density increased from ~ 10^10^ m^-2^ to ~ 7 × 10^13^ m^-2^.^13^ Using a similar approach, Preuß et al. reported the indentation fracture resistance of single-crystal KNbO_3_ was enhanced about ~ 2.8 times with the dislocation densities increased from ~ 2 × 10^11^ m^-2^ up to ~ 10^14^ m^-2^.^14^ These studies on dislocation-mediated plasticity and crack formation assessment mainly focused on mesoscale mechanical properties owing to the fast and simple operation of Vickers indentation. However, current studies on dislocation-based toughening and crack suppression behavior lack more localized, namely, nano-/microscale studies, which are more relevant for electronic device design. To this end, nanoindentation offers an excellent testing platform for probing local volume deformation behavior (with a probing depth of several hundreds of nanometers), which facilitates the direct assessment of the mechanical reliability of electronic devices.^15,16^

In this study, we use nanoindentation tests on perovskite SrTiO_3_ single crystal as a model material to investigate the role of high-density dislocations in toughening and strengthening. SrTiO_3_ has been widely used as a substrate for oxide thin film growth.^17^ Large plastic zones with a length scale of 1–2 mm and dislocation densities spanning over four orders of magnitude (from ~ 10^10^ m^-2^ up to ~ 4.0 × 10^14^ m^-2^) were achieved by Brinell ball cyclic scratching. To set a benchmark with the literature discussed above, Vickers indentation was first used to assess the variation of dislocation densities on the indentation fracture resistance and microhardness. As a key step, nanoindentation was then used to evaluate the crack suppression and hardness. Post-indentation analysis using transmission electron microscopy on both Vickers and nanoindentation imprints reveals the key role of the pre-engineered dislocations on the strengthening and crack suppression of SrTiO_3_ single crystals.

## Materials and Methods

### Material Preparation

SrTiO_3_ is the first perovskite oxide that exhibited room-temperature plasticity during bulk compression (up to ~ 7% plastic strain in the first report).^18^ The room-temperature slip systems are < 110 > {1 $\overline{1 }$ 0} in SrTiO_3_.^19^ The undoped, one-side polished (001) SrTiO_3_ single crystals with a dimension of 5 × 5 × 1 mm^3^ were acquired from Hefei Ruijing Optoelectronics Technology Co., Ltd. (Anhui, China). The surface roughness of the as-polished samples is < 2 nm. The pre-existing dislocation density in the (001) SrTiO_3_ single crystal is ~ 10^10^ m^-2^, with an average spacing of ~ 10 μm between the dislocations, as confirmed by the TEM analysis in our previous work.^20^

### Dislocation Engineering and Characterization

Dislocation imprinting was achieved by surface scratching using a wear testing machine (Rtec, MFT2000, UK) equipped with a spherical indenter (Al_2_O_3_ ruby sphere, 3 mm in diameter), following a similar experimental procedure in previous work.^20^ A normal load of 8 N and a scratching speed of 0.2 mm/s were used for scratching. The scratching track length was set to 1 mm, along the < 100 > direction. Single-direction cyclic scratching for different scratching tracks with various dislocation densities was conducted at intervals of 1×, ×, 20×, and 50× (with × representing the pass number).

Dislocation structures in the scratched tracks were characterized using a transmission electron microscope (TEM, FEI Talos F200X G2, Thermo Fisher Scientific, USA) operating at 200 kV. All the TEM lamellae were lifted in the middle of the scratching tracks along the scratching direction in a dual-beam focused ion beam (FIB) in an SEM (Helios Nanolab 600i, FEI, Hillsboro, USA). Notably, FIB milling with Ga-source can cause artifacts.^21^ To minimize the influence of Ga-implantation, a final FIB-polishing step (with a low current of 41 pA and a voltage of 5 keV) was used to prepare the TEM samples. The annual dark field scanning TEM (ADF-STEM) images were collected with a probe semi-convergence angle of 10.5 mrad and inner and outer semi-collection angles of 23-55 mrad. The dislocation densities in all conditions were then estimated by the line intercept method using the ADF-STEM images.^22^

### Indentation Tests

Microhardness and indentation fracture resistance were assessed using Vickers indentation to directly examine the effects of varied scratching passes (1×, 5×, 20×, and 50×) and dislocation densities. Vickers indentations were performed with loads of 0.01 kg, 0.025 kg, 0.05 kg, 0.1 kg, and 0.2 kg. Lower loads did not produce cracks, while higher loads resulted in larger indent areas and pronounced lateral cracks.^23^ Therefore, an optimal load of 0.2 kg with a holding time of 5 s was chosen to evaluate microhardness and indentation fracture resistance, with each test condition repeated five times. The Vickers indentation spacing is ~ 200 μm. To minimize the influence of lateral cracks, a reference group with a load of 0.05 kg and a holding time of 5 s was used. The indentation-induced cracks in SrTiO_3_ are Palmqvist cracks due to the greater plasticity induced by the pre-existing dislocations in the scratching tracks,^13^ meaning neither the well-known Evans-Charles model or the Lawn-Evans-Marshall model fit the geometry of the induced cracks.^24,25^ Hence, we use the modified Evans-Charles model proposed by Niihara et al. to evaluate the indentation fracture resistance (*K*_*I, IFR*_) using the indentation crack length method according to:^26^

1 $$ {\text{K}}_{{{\text{I}},{\text{ IFR}}}} = 0.035\left( \frac{l}{a} \right)^{ - 0.5} \left( {\frac{E\phi }{H}} \right)^{0.4} \left( {\frac{{Ha^{0.5} }}{\phi }} \right) $$

where $l$ is the length between the indent corner to the crack tip (0.25 ≤ $l$/$a$ ≤ 2.5, which fits our experimental data), *a* is the indent half-diagonal, *E* is the elastic modulus, *H* is the hardness, and $\phi$ is a constant with the value of 3 for SrTiO_3_.^23,26^

Nanoindentation tests were conducted at room temperature using a nanoindentation device (Hysitron TI950, Bruker, USA) to evaluate the nanoindentation hardness and surface crack suppression of the as-scratched sample in a local volume of approximately 1 μm in width (for the nanoindentation imprint). Nanoindentations were made with a diamond Berkovich indenter under a maximum load of 10 mN, resulting in an indentation depth of ~ 180–200 nm. Tests under each condition were repeated at least 10 times to obtain an average value. Indents were spaced 6 μm apart, at over 30 times the indent depth, to avoid the potential overlap of the plastic zones.

Low voltage scanning electron microscopy (LVSEM, Apreo2 S Lovac) operated at 2 keV and 50 pA was then employed to investigate the variation of the nanoindentation imprints with different scratching passes. TEM analysis was performed on the Vickers indents and nanoindents in the reference sample (0×) and 20× scratching samples to examine the dislocation structure evolution.

## Results and Analyses

### Dislocation Characterization

**Fig. 1:**
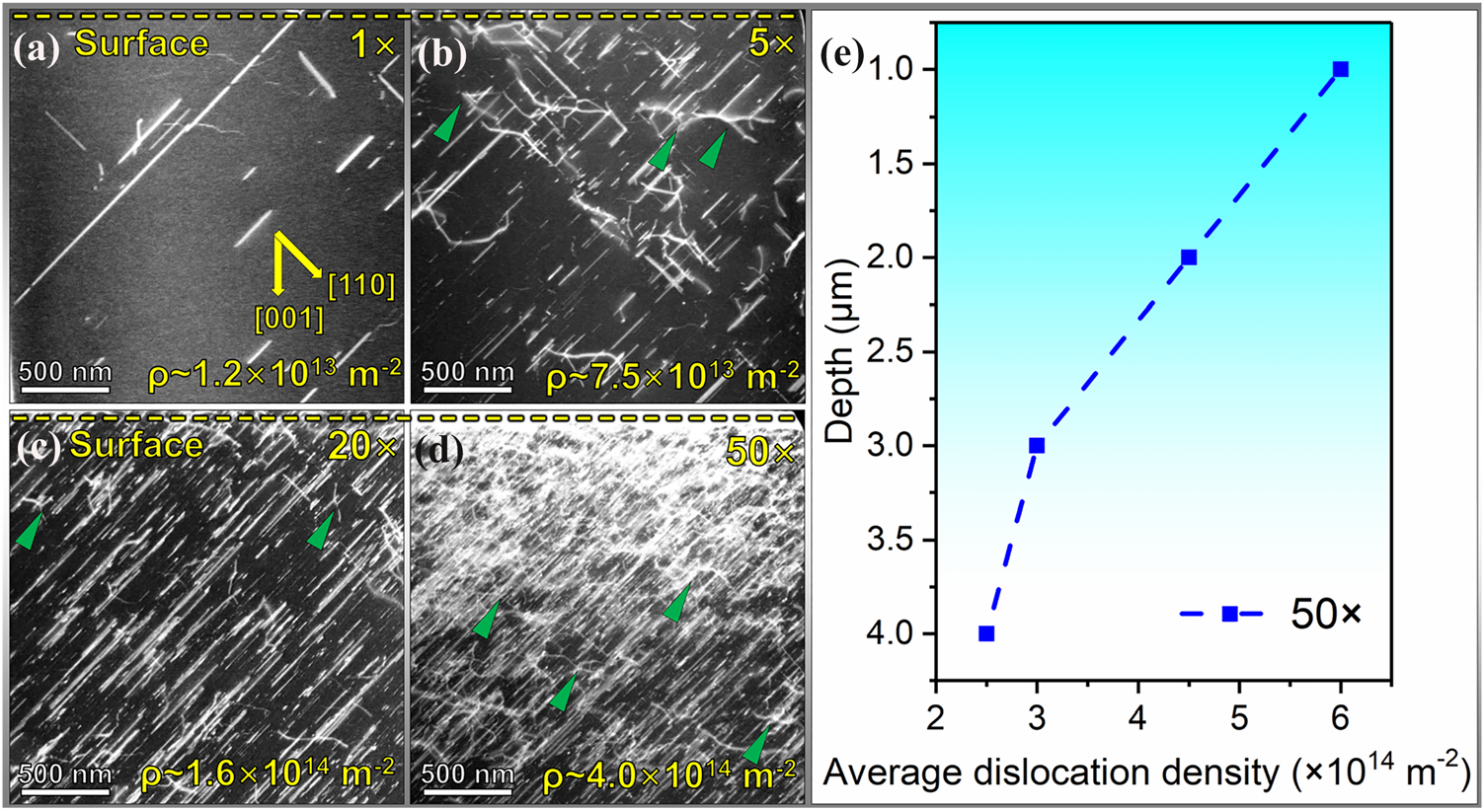
Dislocation structure under the variable number of scratching passes on (001) single-crystal SrTiO_3_. The TEM lamellae were lifted out along the scratching direction, perpendicular to the scratched surface. The crystallographic orientations were marked in (a), and all the TEM images with the same crystallographic orientations were collected along the <001> zone axis. (a) 1× scratching demonstrates the unevenly distributed dislocations; (b) 5× scratching, and (c) 20× scratching shows the increased dislocation densities and uniformly distributed dislocations; (d) 50× scratching displays gradient-distributed dislocations. (e) A gradient of the dislocation density is evident with 50× scratching. Jogs on dislocations are marked by the green triangles.

Figure 1 illustrates the variation of the dislocation density as well as the spatial distribution of the dislocations with the increased number of scratching passes. As revealed in the 1× scratching track, the average dislocation density is approximately 1.2 × 10^13^ m^-2^, with a dislocation spacing of ~ 1 to 2 μm. Another feature from 1× scratching is that the dislocations were distributed unevenly (Fig. 1a). The shear stress (~ 200 MPa, calculated according to Swain and Lawn^27^ based on our experimental data) induced by applying the Brinell ball scratching facilitates the heterogeneous dislocation nucleation and multiplication, similar to the Brinell ball cyclic loading procedure.^20^

As the number of scratching passes increased to 5× and 20×, the dislocation density increased to ~ 7.5 × 10^13^ m^-2^ and ~ 1.6 × 10^14^ m^-2^, and the spatial distribution of the dislocations became more even. Most of the dislocation lines were lying 45° inclined to the (001) surface (Fig. 1b and c), corresponding to the activation of the {110} slip planes.^28^ As the scratching passes increased to 50 × , dislocations were highly tangled and a gradient of the dislocation density from the surface to the interior along the depth became evident, and the average dislocation density in Fig. 1d reached ~ 4 × 10^14^ m^-2^. The dislocation density reached ~ 10^15^ m^2^ in the near-surface layer (yellow dashed line, as captured in Fig. 1d). During the scratching process, dislocations were first generated and multiplied in the near-surface region and propagated to the inner regions, reaching a depth > 100 μm.^20^ As the tangles of dislocations became more severe because of the increased number of scratching passes, dislocation motion can be significantly hindered by the extremely high-density dislocations, leading to aggregation near the surface region. This results in a gradient in the dislocation density ranging from ~ 6 × 10^14^ m^-2^ (surface region) to ~ 3 × 10^14^ m^-2^ (deeper inside the sample), as illustrated in Fig. 1e. Nevertheless, it is worth noting that the sample remains in single-crystal form without grain refinement as in the case of metals.

### Indentation Fracture Resistance

**Fig. 2:**
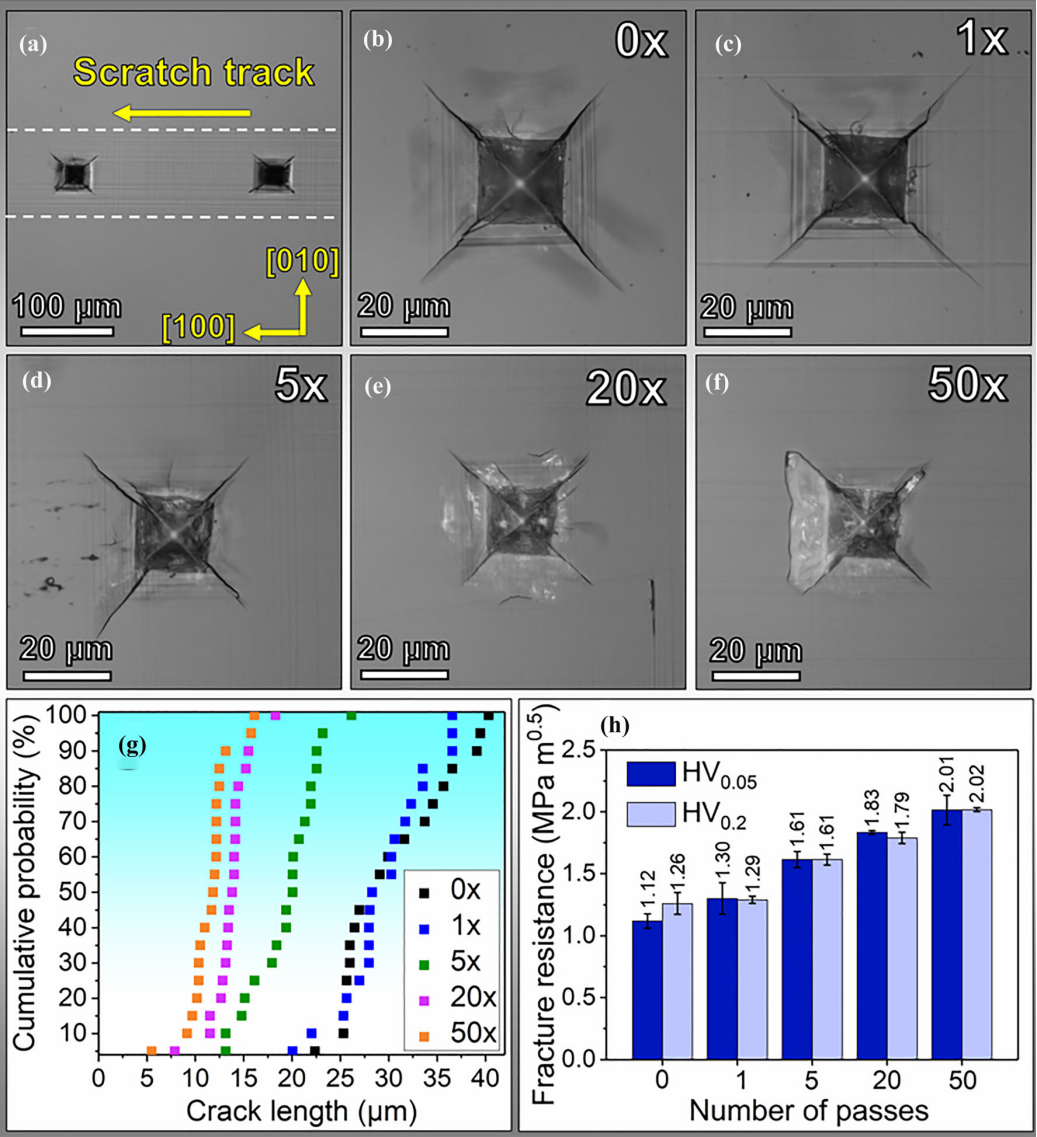
(a)-(f) Representative optical microscopy images reveal the Vickers indentation with 0.2 kg normal load with different scratching passes; (g) cumulative probability of the crack length measured from the four indent corners to the crack tips; (h) comparison of the indentation fracture resistance with different scratching passes under a normal load of 0.05 kg and 0.2 kg.

The representative Vickers indentation imprint morphologies and variations of indentation fracture resistance with increased pre-engineered dislocation densities are shown in Fig. 2a–f. The length of the microcracks along the < 110 > directions, which are the typical cleavage planes for SrTiO_3_ at room temperature,^29^ becomes much shorter with higher densities of pre-induced dislocations. According to the indentation crack length (ICL) method,^30^ this suggests improved indentation fracture resistance (Fig. 2h). Quantitative analysis in Fig. 2g reveals a ~ 36% reduction in crack length from 0× to 5× scratching. Cumulative probability in the *y*-axis is defined as the probability that a random variable will take a value less than or equal to a specific value. Further reduction in crack length was observed with 20× scratching (crack lengths reduced by approximately 55% compared to the reference or 0× sample). No obvious decrease in crack length is observed with 50× scratching compared with 20× scratching. Moreover, signatures of lateral crack formation (the white contrasts around the indents in Fig. 2e and f) were detected for the higher numbers of passes.

To minimize the influence of lateral cracks on the fracture toughness of SrTiO_3_, we compared the change in indentation fracture resistance for two loads (0.2 kg and 0.05 kg, where the formation of lateral cracks is minimized). As summarized in Fig. 2h, Vickers indentations with both loads produced the same effect of increased indentation fracture resistance with increased pre-engineered dislocation densities. Under the indentation load of 0.2 kg, the indentation fracture resistance increased by ~ 60% as the dislocation density reached ~ 4 × 10^14^ m^-2^. This observation is consistent with the previous reports by Porz et al.^2^ and Negm et al.^13^

### Nanoindentation Crack Suppression

**Fig. 3:**
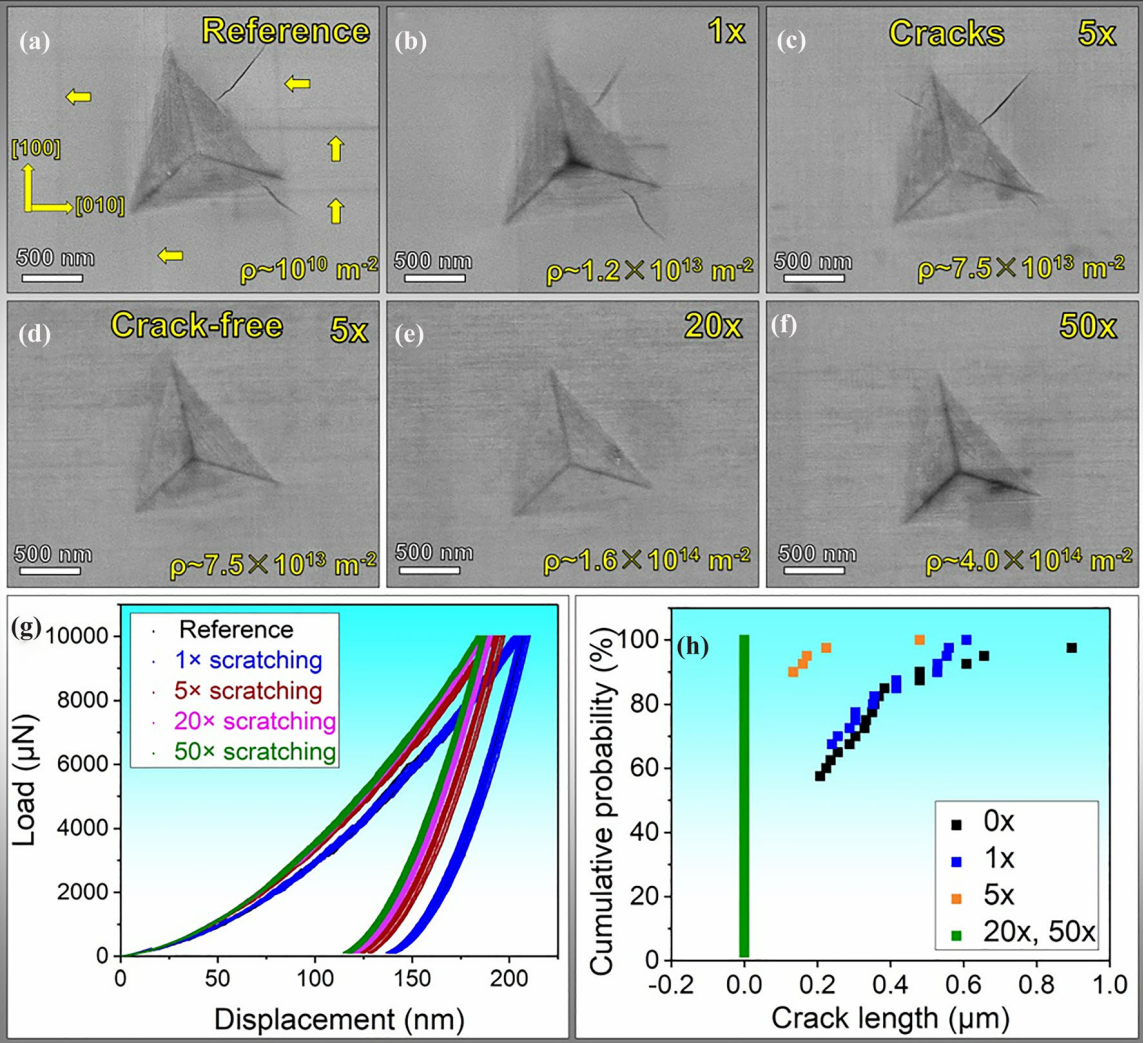
Nanoindentation results of the scratched (001) SrTiO_3_ single crystal. (a-g) SEM images showing the nanoindentation imprints and increased damage tolerance with increased scratching passes/dislocation densities, the yellow arrows in (a) indicate the newly generated slip traces; (g) Load-displacement curves with different scratching passes; (h) cumulative probability of the crack length with different scratching cycle numbers. For each test condition, 20 data points for crack length were collected. The data points corresponding to 0 μm crack length for 1× and 5×  were not visible as they overlapped with the vertical green line for the complete crack suppression for 20× and 50×.

As Vickers indentation generates cracks (Fig. 2b–f), the accuracy of hardness assessment can be affected. Meanwhile, the influence area of Vickers indentation ranges around tens of micrometers, which is not straightforward to investigate the variation of mechanical properties at the submicron-/nanoscale. To this end, we use nanoindentation tests to focus more on crack suppression and nanohardness, which are more relevant for the design of micro-/nanoscale electronic devices such as micro/nano-electro-mechanical systems (MEMS and NEMS). Figure 3 displays the surface morphologies of the nanoindentation imprints with a normal load of 10 mN and the representative load-displacement curves for regions with different numbers of scratching passes. As displayed in Fig. 3a, the main cracks propagated along the < 110 > directions in the reference sample, the same as in the case of Vickers indentation. Besides, slip traces along the [001] and [010] directions were formed after indentation. The dislocation activities in the reference sample during nanoindentation will be discussed in the next section in Fig. 5.

**Fig. 4:**
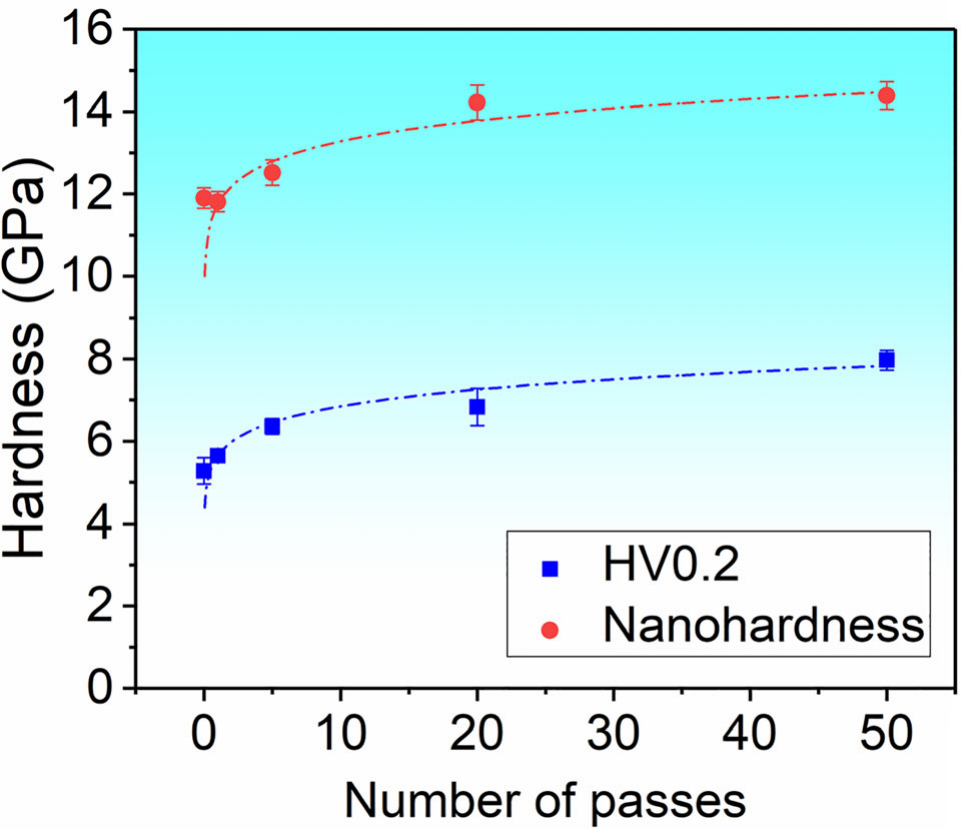
Variation of the Vickers hardness and nanoindentation hardness on the (001) SrTiO_3_ single crystal with different scratching passes/dislocation densities; the dashed curves indicate the tendency of the hardness increase.

The sample with unevenly distributed dislocations with a dislocation density of ~ 1.2 × 10^13^ m^-2^ with 1× scratching also exhibits cracking after indentation. The load-displacement curves of the reference sample and the 1× scratched in Fig. 3g nearly overlapped, indicating the relatively low dislocation density did not have a significant influence on the nanoindentation hardness of the sample (Fig. 4). This is not surprising considering that the size of the nanoindentation imprints is only ~ 1 μm, which is about the same value for the average dislocation spacing in the 1× scratching region (Fig. 1a).

Figure 3h illustrates the variation of crack length tuned by different scratching passes. As the dislocation density increases to ~ 7.5 × 10^13^ m^-2^ (5× scratching), the nanoindentation crack lengths are suppressed by nearly 80% (see the cumulative probability in Fig. 3h). As shown in Fig. 3c, some cracks still formed and propagated along < 110 > direction, but the number of cracks is much less (Fig. 3h). Furthermore, as the dislocation density increases to ~ 1.6 × 10^14^ m^-2^ and ~ 4.0 × 10^14^ m^-2^ with 20× and 50× scratching, respectively, complete crack suppression was achieved, as illustrated in Fig. 3h.

### Indentation Hardness

As summarized in Fig. 4, both Vickers microhardness and nanoindentation hardness increase as the dislocation density increases. The dashed curves in Fig. 4 reveal the tendency of hardness variation affected by the scratching passes and hence dislocation densities. This is in accordance with the decreased areas of Vickers indentation imprint and decreased indentation depth under the same load (Figs. 2 and 3). The Vickers hardness plotted in Fig. 4 was converted from HV to GPa by 1 GPa = 102.04 HV. Note that the Vickers hardness is calculated regardless of crack formation (Fig. 2b–f), which can affect the absolute values of hardness but not the trend in Fig. 4.

With 5× scratching, the Vickers hardness and nanoindentation hardness increased by about 20% and 5%, respectively. As the dislocation density reached ~ 1.6 × 10^14^ m^-2^ and ~ 4.0 × 10^14^ m^-2^, the nanoindentation hardness increased by about 19% and 21%, respectively, with 20× and 50× scratching, and tended to saturate. The Vickers hardness increased by about 30% and 50%, respectively, with 20× and 50× scratching. As the number of the scratching passes increased, dislocation motion was hindered by the enhanced dislocation-dislocation interaction and dislocation-point defect (for instance, oxygen vacancies generated during cyclic scratching) interaction, contributing to hardness increase.^2,31^ The hardness increase also indicates the dislocation work-hardening behavior, which is not surprising in oxide ceramics when high-density dislocations are present.^13,14^ Nevertheless, we note that the hardness in nanoindentation is much higher than the Vickers indentation, with the nanoindentation hardness being ~ 2 times those of Vickers hardness (Fig. 4). This gap indicates an evident size effect.^32–34^

### Post-indentation TEM Characterization

**Fig. 5:**
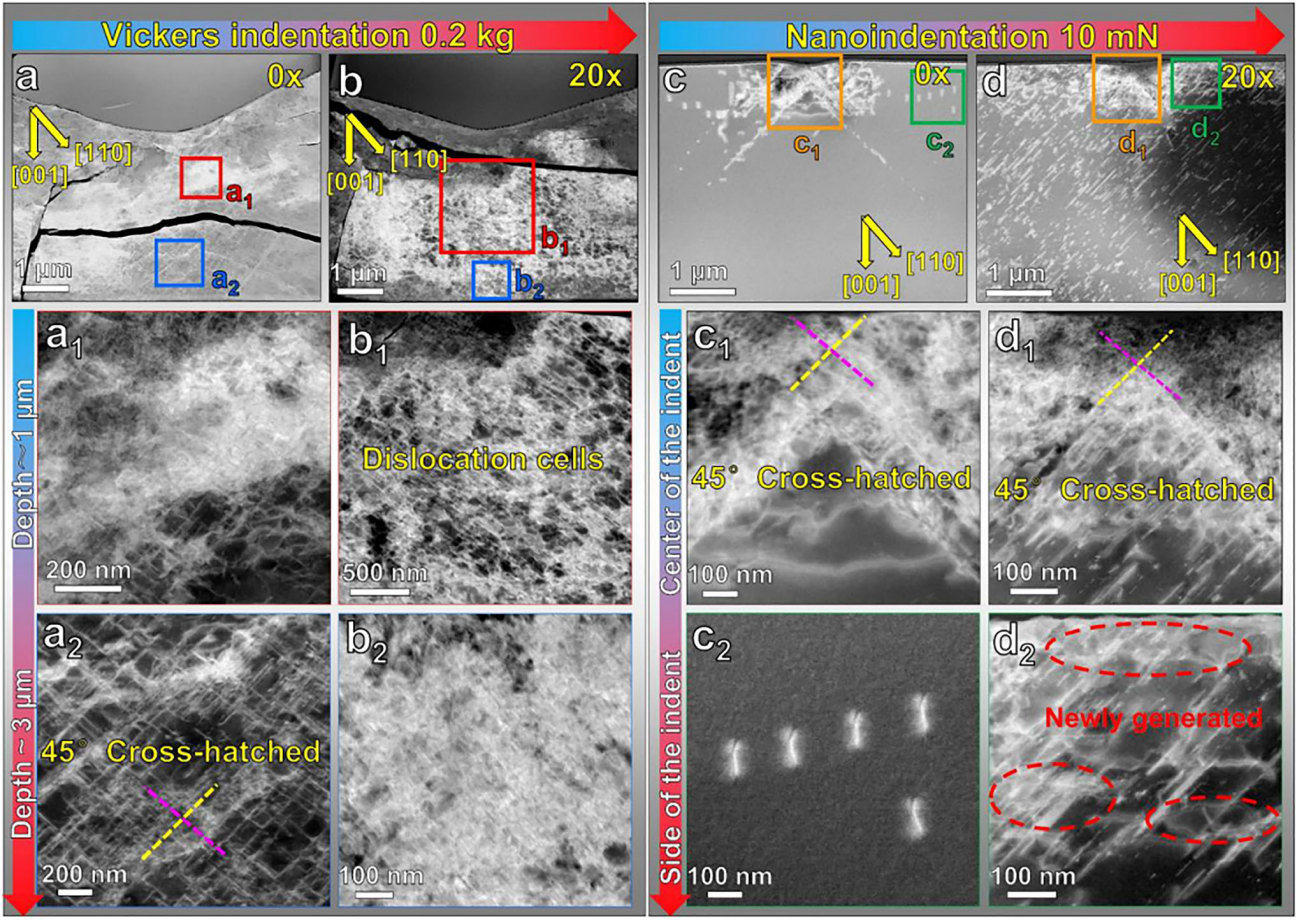
Post-indentation ADF-STEM images reveal the evolution of the dislocation structure after Vickers indentation and nanoindentation. The crystallographic orientations were marked in the figures. All the images are collected along the <001> zone axis. (a) Vickers indentation with a normal load of 0.2 kg in the reference region and (b) in the 20× scratching track; (c) nanoindentation with a normal load of 10 mN in the reference region and (d) in the 20× scratching track.

To reveal the dislocation microstructure evolution beneath the Vickers and nanoindentation imprints, post-indentation TEM analyses were performed on the reference sample and the 20× scratched sample after the indentation tests. All the TEM lamellae were made to reveal the cross-sectional regions (Fig. 5)*.* As demonstrated in Fig. 5a and b, lateral cracks were captured beneath the Vickers indents, which were not detected in the previous optical microscope images (Fig. 2b and e). The lateral crack spread beneath the indents by approximately 1–3 μm. However, it is unclear at this stage whether these lateral cracks were formed because of the indentation or during the FIB milling for the TEM sample preparation. It was reported that the residual stress induced by indentation would be the primary driving force for forming lateral cracks.^35^ Furthermore, lateral cracks can be formed by severe dislocation pileup. The formation of the lateral cracks can also complicate the analysis of the surface crack lengths.^36,37^ In the inner region right beneath the Vickers indenter’s apex, high-density dislocations (Fig. 5a1) are observed at a depth of ~ 1 μm, and 45° cross-hatched dislocations were revealed at a depth of ~ 3 μm in the reference sample (Fig. 5a2**)**. As the pre-engineered dislocation density increased to ~ 1.6 × 10^14^ m^-2^, dislocation cell structures were formed at the depth of ~ 1–3 μm, as illustrated in Fig. 51 and 5b2. These dislocation cells are likely the reasons for the increased Vickers hardness and crack shortening. Dislocation cell structures have been rarely reported in ceramic materials, especially at room temperature. In metals, dislocation cell structures give rise to enhanced hardness by hindering the dislocation motion and dislocation-dislocation interaction.^38^

For nanoindentation, in the reference sample (Fig. 5c), dislocations were imprinted beneath the indents and displayed 45° cross-hatched structures. The nucleation of dislocations was driven by the high shear stress level owning to the sharp indenter tip in nanoindentation.^29,39^ The activation of dislocations corresponds to the formation of slip traces in Fig. 3a. Overall, the dislocations penetrated to a depth of ~ 2 μm. Right beneath the indent within the first ~ 1 μm depth, the dislocations are much more entangled (Fig. 5c1). Due to the lack of pre-existing dislocations, both crack initiation (Fig. 3a) and dislocation generation (Fig. 5c) occurred in the reference sample. Moreover, dislocations also spread laterally much further away from the indent imprint (~2 μm, Fig. 5c2). This is consistent with the observations by Javaid et al.^40^ and Fang et al.,^39^ who used surface chemical etching to reveal that the dislocations induced by nanoindentation in reference samples can spread about 10 times the distance of the depth. As the spacing between the nanoindents in our experiment was set at 30 times the indentation depth, it could thus effectively prevent the overlap of the plastic zones between the two nearest indents. These wide-spreading dislocations in Fig. 5c2 are probably near-screw type dislocations activated for the [011](0 $\overline{1 }$ 1) or [0 $\overline{1 }$ 1](011) slip systems, as visualized in the previous in situ TEM nanoindentation study by Kondo et al.^41^

In contrast to the reference sample, the newly generated dislocations in the 20× scratched region are much more confined within a depth of ~ 1 μm (Fig. 5d), demonstrating direct evidence of back-stress and forest dislocation hardening, which resulted in the increase in nanoindentation hardness in Fig. 4. Worth noting is the further increase in the dislocation density (compared to the as-scratched state) beneath the indent for the 20× sample (Fig. 5c1). This provides direct evidence that, regardless of the pre-engineered high dislocation density with 20× scratching (with a 3-mm Brinell ball indenter), there is still room for further dislocation multiplication to take place under a much higher stress level during the nanoindentation tests (Berkovich indenter). This is a clear indication that dislocation multiplication is strongly dependent on the resolved shear stress levels. As detailed in Fig. 5d2, newly generated dislocations (marked by the red dashed circles) were tangled with the pre-engineered dislocations. Multiplication and generation of new dislocations can consume a large fraction of the input energy during nanoindentation tests, facilitating the suppression of crack initiation and propagation.

## Discussion

The observed increase in dislocation density by cyclic scratching (Fig. 1) is attributed to the dislocation multiplication mechanisms. Johnston and Gilman discussed the dislocation multiplication mechanisms in LiF crystals, which have a cubic structure and share the same room-temperature slip systems with SrTiO_3_.^42^ They proposed that the jog formation on screw dislocations through cross-glide facilitates the multiplication of dislocations. As the number of the scratching passes increases to 50 × , jog formation becomes extremely profuse (see Fig. 1d, marked by the green triangles) because of the significant dislocation interactions, which in turn also generate more jogs because of the intersection of the screw dislocations. In SrTiO_3_, screw-type and mixed-type dislocations are the main dislocation types induced by surface grinding, polishing, and scratching.^3^ Besides, the Frank-Read source mechanism may also contribute to the multiplication of dislocations on a single slip plane.^42,43^ As the number of scratching passes increases, dislocations likely form on multiple parallel planes because of cross-glide mechanisms.^43^ In addition, the dislocations induced during the previous scratching pass can further serve as effective sources for further dislocation multiplication.^3^ These high-density dislocations can facilitate the hardening and toughening behavior in the subsequent Vickers and nanoindentation tests.

The high fracture toughness in metals is mainly achieved by crack-tip dislocation emission and crack-tip blunting.^44^ In contrast, dislocation-based toughening in ceramics has been much less addressed so far because it is difficult to achieve dislocation activities such as dislocation motion and multiplication in most ceramics, particularly at room temperature, except for a few experimental attempts in oxides,^45–47^ alkali halides,^12,48^ and via in situ TEM investigation on Si at small scales.^49^ Here, we obtain a higher fracture toughness indicated by a higher indentation fracture resistance value as the dislocation density is higher with 20× and 50× scratching (~10^14^ m^-2^, with an indentation fracture resistance of ~ 1.8 to 2.0 MPa m^0.5^ in Fig. 2h). In contrast, in the previous studies in SrTiO_3_, the dislocation density is ~ 10^13^ m^-2^ by cyclic indentation or dislocations multiplied by only a surface layer of ~ 5 μm by surface grinding, resulting in an extremely limited area or volume of plastic zones and also lower fracture toughness.^2,13^ However, with the dislocation density increase higher than ~ 10^14^ m^-2^ in this study, a transition in the crack patterns from radial cracks to lateral cracks was observed (Fig. 2e and f). This deserves attention as it suggests that too high a dislocation density may lead to dislocation pileup underneath the indenter to initiate cracks that propagate laterally.

Compared with the most recent study on single-crystal (001) MgO with 0.1 kg load by Preuß et al.,^10^ the Vickers indentation cracks were not completely suppressed on (001) SrTiO_3_ as the load was increased up to 0.1 kg or 0.2 kg. The toughening effect is more pronounced in MgO probably because of a higher density of pre-engineered dislocations (~4 × 10^15^ m^-2^) and the lower lattice friction stress in MgO compared to SrTiO_3_.^10^ Moreover, the dislocation Burgers vector in SrTiO_3_ is twice that in MgO, which means a higher barrier for dislocations to glide in perovskite SrTiO_3_, and the increase in fracture toughness correlates with dislocation activities.^50^ Additionally, the existence of high compressive residual stress can also have a significant influence on preventing crack propagation.^51^

During nanoindentation inside the scratching tracks, further dislocation multiplication and glide facilitate the consumption of the energy and shield of the crack tip,^13^ leading to crack shortening and even crack suppression (Fig. 4). With an increased pre-engineered dislocation density, the number of dislocation sources increases for further dislocation multiplication and motion around the indenter (Fig. 5), leading to additional energy consumption through plastic deformation. This eventually facilitates total suppression of nano-cracks as the dislocation density reaches over ~ 10^14^ m^-2^ during nanoindentation. Notably, this total suppression is also related to the shallower indentation depth of ~ 180 nm in the current nanoindentation tests. In addition, residual stress induced by the cyclic scratching could also contribute to the crack suppression in the Vickers indentations and nanoindentations. To minimize the influence of the residual stress and to clarify a clear effect of the dislocation toughening in SrTiO_3_ as well as other oxides that exhibit dislocation plasticity at room temperature, future work using micro-cantilever bending may be more suitable.^52^

## Conclusion

Contrasting the conventional picture that dislocations are rare in oxides, we used macroscopic Brinell cyclic scratching to achieve large, crack-free zones in millimeter scale with tunable dislocation densities ranging from ~ 10^10^ m^-2^ to ~ 10^14^ m^-2^ on the surface of the (001) SrTiO_3_ single crystal. By increasing the dislocation density, we observed a significant enhancement in the indentation fracture resistance by a factor of 2 (up to ~ 2.0 MPa·m^0.5^). As the dislocation reaches ~ 10^14^ m^-2^, cracks can be completely suppressed within a local volume during nanoindentation with a maximum load up to 10 mN. In addition, both Vickers and nanoindentation hardness increase with the dislocation density, which is governed by the dislocation multiplication and work hardening, as revealed by post-indentation TEM analyses. Hence, the mechanically pre-engineered dislocations serve as effective sources for crack shortening and hardness increase in SrTiO_3_. Our findings highlight the critical role of dislocations in the nano-/micromechanics of functional oxide ceramics and provide insights for designing mechanically reliable electronic devices.
